# Communities of oil palm flower-visiting insects: investigating the covariation of *Elaeidobius kamerunicus* and other dominant species

**DOI:** 10.7717/peerj.7464

**Published:** 2019-08-08

**Authors:** Akhmad Rizali, Bambang Tri Rahardjo, Sri Karindah, Fatma Ramadhani Wahyuningtyas, Nurindah, Bandung Sahari, Yann Clough

**Affiliations:** 1 Department of Plant Pests and Diseases, Brawijaya University, Malang, Indonesia; 2 Indonesian Sweetener and Fiber Crops Research Institute, Malang, Indonesia; 3 PT. Astra Agro Lestari, Jakarta, Indonesia; 4 Centre for Environmental and Climate Research, Lund University, Lund, Sweden

**Keywords:** Scaptodrosophila, Central borneo, Oil palm flower, Sticky trap

## Abstract

Insects visit flowers not only to forage for nectar or pollen but also to search for hosts or prey, and to look for suitable habitats for breeding sites. In oil palm flowers, it has been documented that not all flower-visiting insects are pollinators, but some insects are recognized as predators, parasitoids or saprophages, which may affect the abundance and persistence of the weevil pollinating oil palm, *Elaeidobius kamerunicus*. We studied the community of oil palm flower-visiting insects and investigated the covariation between the abundance *E. kamerunicus* and that of other dominant species. Ecological research was conducted in oil palm plantations with different tree ages in Central Borneo. Our results found that tree age and flower type of oil palm did not influence the abundance and species richness of flower-visiting insects, but significantly affected their species composition. There was a significant positive relationship between the abundance of *E. kamerunicus* and the fly *Scaptodrosophila* sp, indicating that these species covariate in oil palm flowers. These findings suggest that understanding the covariation between *E. kamerunicus* and *Scaptodrosophila* sp may help develop the conservation strategies for *E. kamerunicus* to support the sustainable production of oil palm.

## Introduction

The presence of insects in oil palm flowers is related to their activity to look for nectar or pollen (Lajis, Hussein & Toia, 1985; Syed, 1979) or to search for prey (Hakim et al., 2017) as well as for suitable habitat for breeding sites (Corley & Tinker, 2003; Moore, 2001). The identity of the insects visiting oil palm flowers depends on the geographical region. In Africa, which is the origin area of oil palm plants, the most dominant flower visitors are *Elaeidobius kamerunicus*, *E. plagiatus* and *E. subvittatus* (Coleoptera: Curculionidae): these insects have an important role as a pollinators (Syed, 1979). In South America, the main pollinator of oil palm is *Mystrops costaricensis* (Nitidulidae), while in Asia it is *Thrips hawaiiensis* (Corley & Tinker, 2003). In Indonesia, since the introduction of *E. kamerunicus* in 1983, this weevil has become the most abundant oil palm flower-visiting insect, and its presence has been an important contribution to increasing fruit set of oil palm (Susanto, Purba & Prasetyo, 2007).

*E. kamerunicus* is well-adapted to wet tropical climates and found with high abundance in oil palm flowers in Indonesia (Prasetyo & Susanto, 2012). *E. kamerunicus* feeds and breeds in male inflorescences of oil palm (Corley & Tinker, 2003; Syed, 1979). Pollination occurs when *E. kamerunicus*, unintentionally carrying the pollen from male inflorescences on their elytra, visit female inflorescences. The weevil visits the receptive female inflorescences due to the attractive effect of estragole, a volatile compound released by female flowers that is similar to volatile compounds released by male flowers (Susanto, Purba & Prasetyo, 2007).

The production of oil palm, in terms of weight of bunches and the number of fruits set, has increased after the introduction of *E. kamerunicus* to Indonesia (Lubis, Sudarjat & Dono, 2017). A high oil palm fruit set (i.e., above 75%) requires a population of at least 20,000 *E. kamerunicus* individuals per hectare (Donough, Chew & Law, 1996). At present, oil palm cultivation is experiencing problems with decreasing fruit set (Prasetyo, Purba & Susanto, 2014; Teo, 2015). This is likely due to factors such as side effects of insecticide applications or increases in natural enemies of *E. kamerunicus* such as rats (Bessou et al., 2017), nematodes (Poinar et al., 2002), mites (Krantz & Poinar, 2004) or other predators (Hakim et al., 2017). For this reason, efforts are needed to increase the population of *E. kamerunicus* and to maintain the population above the minimum threshold needed to effectively pollinate the oil palm (Kahono et al., 2012). For instance, the population of *E. kamerunicus* can be increased in the field using the hatch and carry method (Prasetyo, Purba & Susanto, 2014). Further research is needed to better understand the drivers that affect the population of *E. kamerunicus*; it has, for instance, been shown that factors such as the tree age of palm oil (Rahardjo et al., 2018) as well as interactions with other flower-visiting insects (Hakim et al., 2017; Syed, 1979) affect the population of *E. kamerunicus* in the field.

Understanding the interaction between insect pollinators and other flower-visiting insects (anthophiles) is an importance aspect in ecosystem functioning and agricultural production (Kevan, 2008). As relatively primitive insect pollinators, Coleoptera and Diptera were documented on the fossil record as pollen vectors (Bernhardt, 2000; Kevan & Baker, 1983; Labandeira, 1998) and in recent times both insect groups can be found on the same plant, for instance in oil palm (Syed, 1979). Drosophilid flies (Diptera: Drosophilidae) are highly diverse as flower visitors and derive carbohydrate and utilize yeasts for their nutrition at flowers. Some species of curculionid beetles (Coleoptera: Curculionidae) were also reported to eat decomposed flowers (Moore, 2001; Syed, 1982). The presence of drosophilids and curculionids in the same flower may be associated with competition for resources, alternatively they may covary without any interaction.

In this research, we studied the community of oil palm flower-visiting insects in oil palm plantation in Central Borneo, Indonesia. We addressed the following questions: (i) which factors affect the communities of flower-visiting insects in oil palm plantations, and (ii) is there a relationship between the abundance of *E. kamerunicus* and that of other dominant species, while controlling for other factors? Information about covariation of flower-visiting insects is needed to understand ecosystem functioning and to develop a conservation strategy for pollinators of oil palm in Indonesia.

## Materials & Methods

### Research site and determination of sampling units

**Figure 1:**
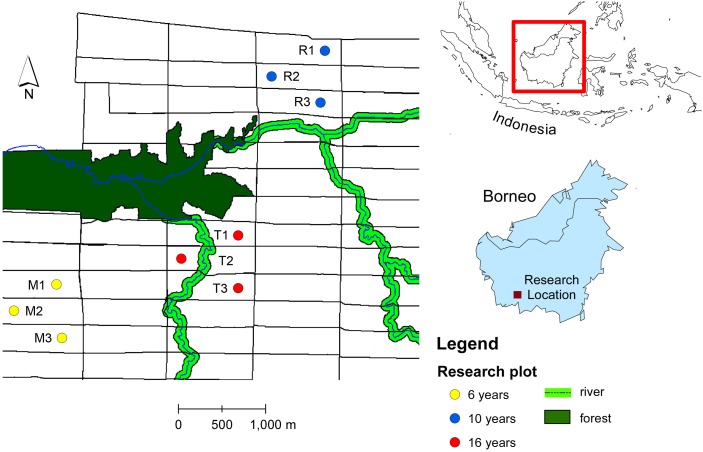
Map of study sites in oil plantation in Central Borneo, Indonesia. The letter and number refer to plot code listed in Table 1. Plots were selected in different tree age (6, 10 and 16 years) that located in different block of oil palm field with size of each block 300 m × 1,000 m (30 ha) and each block have the same tree age.

The ecological research was conducted in an oil palm plantation in Pangkalan Lada, Central Borneo, Indonesia. The tree age ranges from 4 years to 20 years. The oil palm with the same age were planted in a block with size 300 m × 1,000 m (30 ha) (Fig. 1). We chose productive oil palm plots with different tree ages: 6, 10 and 16 years-old. Within each age group, we selected three oil palm fields from different blocks. Within each oil palm field we selected a sampling plot. The sampling plot was a hundred oil palm trees (10 × 10 trees). The number of oil palm inflorescences varies in space and time due to environmental and plant genetic factors (Adam et al., 2011). To standardize the sampling unit, we sampled two anthesizing male inflorescences and two receptive female inflorescences in each plot, as this was the lowest number of oil palm flowers recorded from all plots across different tree ages (Table 1). The oil palm flower data were obtained by counting the number of anthesizing male and receptive female inflorescences in each plot before sampling. Every month, the number of male flowers ranges from 5–8 inflorescences per hundred trees, while female flowers range from 2–5 inflorescences per hundred trees.

**Table 1:** Plot characteristics of nine studied oil palm fields with different tree age, and diversity of oil palm flower-visiting insects both from male and female inflorescences. Number of mature inflorescence is the average of three sampling times in different months (*n* = 3). The numbers of insects are the total for six different inflorescences that two measured for each of three months. S: species richness, N: number of individuals.

Tree age (year)	Plot code	No. of mature inflorescence (mean ± SD)		Average of tree height (m) (*n* = 25)	Light intensity (lux) (*n* = 15)	Vegetation diversity (*n* = 10)	Insect diversity					
							Male		Female		Total	
		Male	Female				S	N	S	N	S	N
6	M1	7.7 ± 1.5	4.7 ± 0.6	2.7 ± 0.3	293 ± 52	15	53	7,797	38	8,066	68	15,863
	M2	6.7 ± 1.5	3.7 ± 0.6	3.5 ± 0.3	310 ± 61	14	43	7,384	55	6,511	75	13,895
	M3	7.0 ± 1.7	3.7 ± 1.2	4.0 ± 0.4	311 ± 45	14	70	11,513	69	15,264	106	26,777
10	R1	6.3 ± 0.6	4.0 ± 0.0	7.2 ± 0.4	255 ± 44	9	57	12,122	71	5,372	101	17,494
	R2	6.3 ± 1.5	4.0 ± 1.0	7.3 ± 0.6	259 ± 43	16	53	3,153	46	3,723	74	6,876
	R3	5.7 ± 1.2	3.0 ± 0.0	6.8 ± 0.9	263 ± 46	9	49	6,746	55	10,115	74	16,861
16	T1	6.0 ± 1.7	2.0 ± 0.0	9.1 ± 0.5	214 ± 25	19	46	14,802	63	11,580	79	26,382
	T2	7.3 ± 0.6	2.3 ± 0.6	9.6 ± 0.6	227 ± 31	20	47	14,855	48	22,896	69	37,751
	T3	7.3 ± 0.6	2.0 ± 0.0	10.1 ± 0.7	236 ± 34	12	50	10,995	39	8,680	65	19,675
						Total	199	89,367	198	92,207	275	181,574

We also measured the plot characteristics including tree height, light intensity and understorey vegetation diversity on each plot. Light intensity was measured using a lux meter that was set up close to male and female inflorescences. While the observation of understorey vegetation was done in 10 randomly placed 1 × 1 m quadrats. In all blocks, the management of understorey vegetation was managed by grazing with cows, without herbicide application. The diversity of understorey vegetation at each point was noted and the specimen samples were taken or photographed to be identified in the laboratory. Identification of vegetation specimens was conducted using the reference of Xu & Zhou (2017).

### Sampling and identification of oil palm flower-visiting insects

The sampling of oil palm flower-visiting insects was done by installing a sticky trap in two male and two female inflorescences in each plot. The sticky traps were made from transparent plastic with size 15 cm ×10 cm and smeared with an adhesive material (rat glue). Five traps were mounted circularly covering all parts of an inflorescence and were installed during the day (07.00 am–16.00 pm) and the night (16.00 pm–07.00 am) to collect flower visitors both of diurnal and nocturnal insects. Trapped insects then were preserved using 70% alcohol for further sorting and identification in the laboratory. In each plot, insect sampling was conducted every month in different inflorescences, during three months from March to May 2016.

Specimens of flower-visiting insects were initially sorted to order and family level using the identification books such as Borror, Triplehorn & Johnson (1996), Goulet & Huber (1993) and McAlpine (1987). Afterwards, each order or family of insects was then identified to morphospecies level based on the differences of morphological characters and if possible until genera level especially for ants (using Bolton, 1994) and flies (using Bock, 1976).

### Data analysis

The difference of dominant insect abundance between male and female inflorescences was tested using analysis of variance (ANOVA). Effect of environmental factors on the richness and abundance of flower-visiting insects was analyzed by fitting a generalized linear model (GLM) without interactions (Zuur et al., 2009) and using a quasiPoisson distribution to account for overdispersion. Explanatory variables included tree age of oil palm, flower type (male/female), and vegetation diversity. We excluded tree height (Pearson’s *r* = 0.962, *P* < 0.001) and light intensity (Pearson’s r = −0.955, *P* < 0.001) due to strong correlation with tree age of oil palm.

The effect of environmental factors on species composition of flower-visiting insects was analyzed by canonical correspondence analysis (CCA) and continued using forward selection with 1,000 permutations. In addition, pairwise test from analysis of similarity (ANOSIM) with the Bray-Curtis index was also used to compare insect species composition between different tree ages of oil palm (Legendre & Legendre, 1998).

Covariation between *E. kamerunicus* and other dominant insect species was analyzed using GLM with the abundance of dominant species (*Scaptodrosophila* sp, *Pheidole* sp and *Gelechiidae* sp), tree age, flower type of oil palm, and vegetation diversity as explanatory variables.

All analyzes were performed using R statistical software (R Core Team, 2018) and utilizing the vegan package for CCA and ANOSIM (Oksanen et al., 2015).

## Results

### Diversity and species composition of oil palm flower-visiting insects

**Figure 2:**
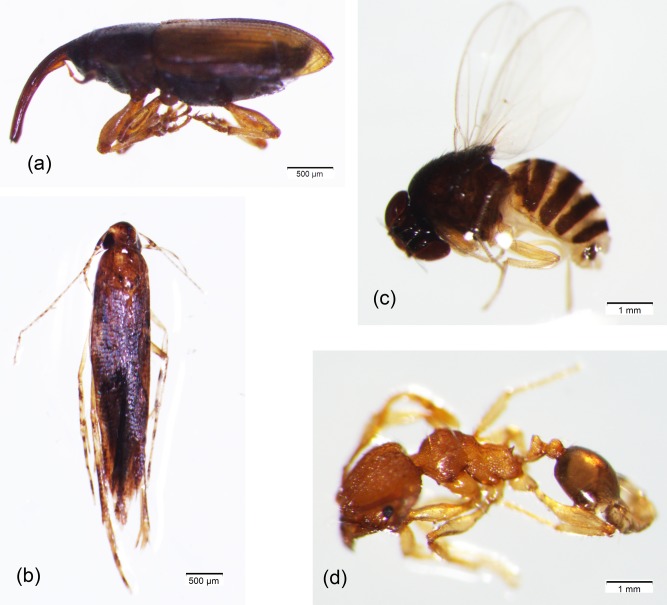
The most dominant species of flower-visiting insects in oil palm plantation in Central Borneo, Indonesia. (A) *Elaeidobius kamerunicus*, (B) *Gelechiidae* sp, (C) *Scaptodrosophila* sp, and (D) *Pheidole* sp.

The diversity of oil palm flower-visiting insects recorded across all plots was 275 species from 10 orders and 181,574 individuals (Tables 1 and 2). The Coleoptera were most abundant and dominated by *E. kamerunicus* (Fig. 2A). Other dominant insects were Diptera, dominated by *Scaptodrosophila* sp, Hymenoptera which were dominated by ants (*Pheidole* sp) and Lepidoptera which were dominated by a moth species (*Gelechiidae* sp) (Table 2, Figs. 2B–2D). The abundance of Coleoptera (*F*_1,52_ = 0.342, *P* = 0.561) and Lepidoptera (*F*_1,52_ = 0.012, *P* = 0.914) were not different between male and female inflorescences. In contrast, the abundance of Diptera was significantly higher in male inflorescences (*F*_1,52_ = 35.490, *P* < 0.001), while Hymenoptera were more abundant in female inflorescences (*F*_1,52_ = 4.057, *P* = 0.049).

**Table 2:** Species richness (S) and number of individuals (N) of each order of flower-visiting insects in male and female inflorescences from all plots.

No	Order	Male		Female		Total		Dominant species (% of N total)
		S	N	S	N	S	N	
1.	Blattodea	1	7	1	3	1	10	
2.	Coleoptera	20	75,320	16	84,098	28	159,418	*Elaeidobius kamerunicus* (99.9%)
3.	Dermaptera	2	5	4	8	5	13	
4.	Diptera	97	11,603	81	4,282	121	15,885	*Scaptodrosophila* sp (89.1%)
5.	Hemiptera	6	18	7	9	8	27	
6.	Homoptera	10	11	8	9	13	20	
7.	Hymenoptera	47	565	62	1,989	78	2,554	*Pheidole* sp (55.9%)
8.	Lepidoptera	6	1,790	8	1,755	10	3,545	*Gelechiidae* sp (94.4%)
9.	Mantodea	1	1	1	1	1	2	
10.	Orthoptera	9	47	10	53	10	100	

The results of GLM showed that tree age, flower type of oil palm and vegetation diversity did not influence the species richness and abundance of flower-visiting insects (Table 3). In addition, the CCA revealed that the species composition of flower-visiting insects was significantly affected by flower type and tree age of oil palm (Table 4). The ANOSIM results also proved that the composition of flower-visiting insects differed between flower type (*R* = 0.039, *P* = 0.046) and tree age (*R* = 0.113, *P* = 0.001). Species composition of flower-visiting insects was significantly different between palms 6 and 16 years old, as well as between palms 10 and 16 years old, but not between palms 6 and 10 years-old (Table 5).

**Table 3:** Generalized linear models relating species richness and abundance of flower-visiting insects to tree age, flower type and vegetation diversity as predictors.

Variable	Species richness			Abundance		
	Estimate	SE	*P*	Estimate	SE	*P*
(Intercept)	3.470	0.151	<0.001	6.901	0.324	<0.001
Tree age	−0.011	0.008	0.186	0.034	0.020	0.089
Vegetation diversity	−0.006	0.009	0.506	0.033	0.021	0.129
Flower type (male)	−0.027	0.063	0.669	−0.031	0.141	0.825
Plot (2)	0.154	0.079	0.057	0.458	0.186	0.018
Plot (3)	0.191	0.078	0.019	0.486	0.185	0.012
Month (2)	−0.196	0.077	0.014	−0.187	0.185	0.318
Month (3)	−0.141	0.076	0.068	0.267	0.166	0.114

**Table 4:** Effects of explanatory variables related to flower type, tree age, light intensity and vegetation diversity on oil palm flower-visiting species composition in oil palm plantation. Results of forward selection procedure within a canonical correspondence analysis using the ordistep method with 1,000 permutations.

Variable	DF	AIC	*F*	*P*-value
Flower type	1	393.52	3.926	0.005
Tree age	1	395.46	1.950	0.020
Vegetation diversity	1	396.22	1.197	0.135

**Table 5:** One-way analyses of similarity (ANOSIM) testing for differences in oil palm flower-visiting insect species composition between oil palm tree ages.

Tree age	*R*	*P*-value
6 years vs. 10 years	0.052	0.074
6 years vs. 16 years	0.076	0.037
10 years vs. 16 years	0.206	0.001

### Covariation in abundance of *E. kamerunicus* and other dominant species

We focused on the covariation of *E. kamerunicus* with the other dominant species in the flower-visiting community: *Scaptodrosophila* sp, *Pheidole* sp and *Gelechiidae* sp (Table 2). The results of GLM showed that the abundance of *E. kamerunicus* was positively affected by abundance of *Scaptodrosophila* sp (*P* = 0.001), vegetation diversity (*P* = 0.007) and female flower type (*P* = 0.008) (Table 6). The same pattern for abundance of *Scaptodrosophila* sp was also positively affected by abundance of *E. kamerunicus* (*P* = 0.001), vegetation diversity (*P* = 0.009) and flower type (*P* < 0.001) (Table 6).

**Table 6:** Generalized linear models relating abundance of *E. kamerunicus* and *Scaptodrosophila* sp to tree age, flower type, vegetation diversity, and abundance of dominant species as predictors. The dominant species are *E. kamerunicus*, *Scaptodrosophila* sp, *Pheidole* sp, and *Gelechiidae* sp.

Variable	*E. kamerunicus*			*Scaptodrosophila* sp		
	Estimate	SE	*P*	Estimate	SE	*P*
(Intercept)	6.144	0.386	<0.001	4.910	0.382	<0.001
*Scaptodrosophila* sp	0.002	0.000	0.001			
*E. kamerunichus*				0.000	0.000	0.001
*Pheidole* sp	0.000	0.001	0.892	0.001	0.001	0.624
*Gelechiidae* sp	0.002	0.002	0.202	0.000	0.002	0.997
Tree age	0.006	0.022	0.798	0.028	0.022	0.196
Vegetation diversity	0.064	0.022	0.007	−0.056	0.020	0.009
Flower type (male)	−0.537	0.193	0.008	1.158	0.164	0.000
Plot (2)	0.366	0.208	0.086	0.172	0.191	0.374
Plot (3)	0.655	0.194	0.002	−0.251	0.191	0.196
Month (2)	−0.148	0.189	0.438	−0.030	0.172	0.864
Month (3)	0.360	0.164	0.034	−0.151	0.172	0.384

## Discussion

The most dominant oil palm flower-visiting insect in oil palm plantations in Central Borneo is *E. kamerunicus*. An introduced species, this weevil has adapted well to Indonesian oil palm plantations, yet their populations have been shown to be prone to decline (Prasetyo, Purba & Susanto, 2014). The second dominant species was *Scaptodrosophila* sp, a member of the drosophilid flies that is widespread in tropical Asia and known to feeding and breeding sites in fruit, flowers and leaves (Bock & Parsons, 1978). In oil palm plantations, *Scaptodrosophila* sp was found in high abundance in male inflorescences. It indicated that male inflorescence of oil palm contains food sources and suitable sites for breeding of *Scaptodrosophila* sp. Barker (2005) showed that species of Scaptodrosophila are restricted to flowers of certain plant species for feeding and breeding.

The ant species *Pheidole* sp was also found dominant in oil palm flowers. Kahono et al. (2012) reported that ants actively visit the flowers of oil palm both on receptive female inflorescence and anthesizing male inflorescence. The role of ants in oil palm flowers may include foraging for nectar or for prey, but this has to our knowledge never been investigated further. Nectar is an attractant for flower visiting insects including pollinators, herbivores, predators or parasitoids (Strauss & Whittall, 2006). In addition, a moth morphospecies (*Gelechiidae* sp) was also found dominant in oil palm flowers. As nocturnal insects, moths visit oil palm flowers during the night to find flower nectar and their feeding activity also have a contribution to pollination (Moore, 2001). However, *E. kamerunicus* is the most effective pollinator of oil palm due to its ability to carry many pollen grains compared with other *Elaeidobius* species (Kouakou et al., 2014) and other potential pollinators such as the moths *Pyroderces* sp. (Momphidae) and *Thrips hawaiiensis* (Corley & Tinker, 2003; Moore, 2001). Male weevils carry more pollen than female weevils because they have a larger body size and more setae (Moore, 2001). Surprisingly, *T. hawaiiensis*, as a former potential pollinator in Asia (Corley & Tinker, 2003) was not recorded in this research.

In this study, we found that tree age of oil palm did not affect the species richness and abundance of flower-visiting insects. However, increasing tree age affected the species composition of flower-visiting insects. As a consequence of increasing tree age, the architecture of oil palm plants such as tree height and a canopy is also changing. This may increase the availability of nest sites and microhabitats for insects, thus shaping the diversity as well as species composition of insects in oil palm plantation. Research by Sahari (2012) revealed that insects, and especially parasitoid wasps, were more diverse in the open canopy with more sunlight. Open canopy also facilitates the diversity of understorey vegetation especially flowering plants that provide alternative habitat and food source for pollinator insects (Klein, Steffan-Dewenter & Tscharntke, 2003) as well as natural enemies (Perovic et al., 2010). In cacao agroforestry system, increasing age of cacao tree changed the architecture of cacao tree as well as shade trees and affected the species composition of ants (Rizali et al., 2013).

The difference of flower types also affected the species composition of oil palm flower-visiting insects. Male and female inflorescences have different structural morphologies in which male flower have pollen and nectar and different volatile compounds compared to the female flower; therefore, it affects preference for the visiting insects (Moore, 2001; Syed, 1979). However, the receptive female flower of oil palm produces estragole, a volatile compound that is also produced by the male flower, and that attracts *E. kamerunicus* to visit despite absence of food or nesting site in female flowers (Susanto, Purba & Prasetyo, 2007).

The analysis of the relationship between *E. kamerunicus* and other dominant species, revealed that the abundance of *E. kamerunicus* is positively correlated to the abundance of *Scaptodrosophila* sp, while controlling for environmental variables. *Scaptodrosophila* sp, like *E. kamerunicus*, is arguably utilizing male flower of oil palm for feeding and breeding sites, while other dominant insects, ants and moths were merely looking for nectar. The difference between *E. kamerunicus* and *Scaptodrosophila* sp was that the abundance of *Scaptodrosophila* sp was higher in male than in female inflorescences, with *E. kamerunicus* showing no such difference. The covariation between *E. kamerunicus* and *Scaptodrosophila* sp in oil palm flowers was presumably related to the similar behaviour of both species as fungus-eating insects (mycophagous). Coexistence between fungus-eating insects is well known from other systems (Kadowaki, 2010). In Africa, *E. kamerunicus* may coexist with other fungus weevils such as Nitidulidae and Mycetophagidae (Syed, 1979) which have an important role in decomposition processes. Biological studies showed that *E. kamerunicus* do not eat pollen, the adults feed only the inside part of a male flower of oil palm and larvae develop on decomposed flowers (Moore, 2001; Syed, 1982). Feeding activity of the weevils may facilitate the growth of fungi and bacteria for the decomposition process of waste food material. The presence of fungi and bacteria may attract *Scaptodrosophila* sp to visit the oil palm flowers for feeding and breeding (Jacome et al., 1995).

Bacteria and fungi have an important role for drosophilid flies as food sources and increasing their fitness. Therefore, drosophilids transfer both bacteria and fungi during mating. Bacteria are the most important microbes for decomposition, while fungi (yeasts) play a role in fermentation (Markow & O’Grady, 2008). Drosophilids are attracted to visit and oviposit by ethanol (Hoffmann & Parsons, 1984) which may be produced by yeast during the decomposition process of waste material that has been utilized by *E. kamerunicus*. In addition, drosophilids also deposit bacteria and fungi in breeding sites during eggs laying to increase the food resource for larvae.

## Conclusions

This study found that the abundance of *E. kamerunicus* is not only positively related to the vegetation diversity within oil palm plantation, but also to the abundance of *Scaptodrosophila* sp. Although the mechanism is uncertain yet, it is a possibility that *E. kamerunicus* has mutualistic interaction with *Scaptodrosophila* sp. Further study is needed to investigate the interaction mechanism between *E. kamerunicus* and *Scaptodrosophila* sp as well as their symbiont microbes. We believe that understanding those interactions will provide significant benefit for conservation and management strategy of *E. kamerunicus* in oil palm plantation (Li et al., 2019), beside understanding the biology of *E. kamerunicus* (Tuo, Koua & Hala, 2011), releasing *E. kamerunicus* to increase pollination (Prasetyo, Purba & Susanto, 2014) as well as controlling predators and other natural enemies of *E. kamerunicus* (Hakim et al., 2017).

##  Supplemental Information

10.7717/peerj.7464/supp-1 Dataset S1 Diversity of flower-visiting insects in oil palm plantation in Central Borneo, Indonesia that collected from both male and female inflorescence in different tree age of oil palm (6, 10, 16 years) during 3 months of sampling

10.7717/peerj.7464/supp-2 Figure S1 Species accumulation curves of flower-visiting insects between (a) all plots and different tree ages, (b) plots with tree age 6 years, (c) plots with tree age 10 years and (d) plots with tree age 16 years.

## Additional Information and Declarations

### Data Availability

The following information was supplied regarding data availability: The raw data of flower-visiting insects are available in the Supplemental File.

## References

[ref-1] Adam H, Collin M, Richaud F, Beule T, Cros D, Omore A, Nodichao L, Nouy B, Tregear JW (2011). Environmental regulation of sex determination in oil palm: current knowledge and insights from other species. Annals of Botany.

[ref-2] Barker JSF (2005). Population structure and host-plant specialization in two *Scaptodrosophila* flower-breeding species. Heredity.

[ref-3] Bernhardt P (2000). Convergent evolution and adaptive radiation of beetle-pollinated angiosperms. Plant Systematics and Evolution.

[ref-4] Bessou C, Verwilghen A, Beaudoin-Ollivier L, Marichal R, Ollivier J, Baron V, Bonneau X, Carron M-P, Snoeck D, Naim M, Ketuk Aryawan AA, Raoul F, Giraudoux P, Surya E, Sihombing E, Caliman J-P (2017). Agroecological practices in oil palm plantations: examples from the field. Oilseeds & Fats Crops and Lipids (OCL).

[ref-5] Bock IR (1976). Drosophilidae of Australia: Drosophila (Insecta: Diptera). Australian Journal of Zoology.

[ref-6] Bock IR, Parsons PA (1978). The subgenus *Scaptodrosophila* (Diptera: Drosophilidae). Systematic Entomology.

[ref-7] Bolton B (1994). Identification guide to the ant genera of the world.

[ref-8] Borror D, Triplehorn CH, Johnson NF (1996). An introduction to the study of insects.

[ref-9] Corley RHV, Tinker PB (2003). The oil palm.

[ref-10] Donough CR, Chew KW, Law IH (1996). Effect of fruit set on OER and KER: results from studies at Pamol Estates (Sabah) Sdn Bhd. The Planter.

[ref-11] Goulet H, Huber JT (1993). Hymenoptera of the world: an identification guide to families.

[ref-12] Hakim L, Nasir D, Ghani IA, Hazmi I (2017). The potential natural predators of *Elaeidobius kamerunicus* Faust, 1878 (Coleoptera: Curculionidae) in Malaysia. Serangga.

[ref-13] Hoffmann AA, Parsons PA (1984). Olfactory response and resource utilization in Drosophila: interspecific comparisons. Biological Journal of the Linnean Sociey.

[ref-14] Jacome I, Aluja M, Liedo P, Nestel D (1995). The influence of adult diet and age on lipid reserve in the tropical fruit fly, *Anastrepha serpentina* (Diptera: Tephritidae). Journal of Insect Physiology.

[ref-15] Kadowaki K (2010). Species coexistence patterns in a mycophagous insect community inhabiting the wood-decaying bracket fungus *Cryptoporus volvatus* (Polyporaceae: Basidiomycota). European Journal of Entomology.

[ref-16] Kahono S, Lupiyaningdyah P, Erniwati, Nugroho H (2012). Potensi dan pemanfaatan serangga penyerbuk untuk meningkatkan produksi kelapa sawit di perkebunan kelapa sawit Desa Api-api. Kecamatan Waru, Kabupaten Penajam Paser Utara, Kalimantan Timur. Jurnal Zoo Indonesia.

[ref-17] Kevan PG, Capinera JL (2008). Pollination and flower visitation. Encyclopedia of entomology.

[ref-18] Kevan PG, Baker HG (1983). Insects as flower visitors and pollinators. Annual Review of Entomology.

[ref-19] Klein A-M, Steffan-Dewenter I, Tscharntke T (2003). Pollination of *Coffea canephora* in relation to local and regional agroforestry management. Journal of Applied Ecology.

[ref-20] Kouakou M, Hala NK, Akpesse AAM, Tuo Y, Dagnogo M, Konan KE, Koua HK (2014). Comparative efficacy of *Elaeidobius kamerunicus, E. plagiatus, E. subvittatus* (Coleoptera: Curculionidae) and *Microporum* spp. (Coleoptera: Nitidulidae) in the pollination of oil palm (*Elaeis guineensis*). Journal of Experimental Biology and Agricultural Sciences.

[ref-21] Krantz GW, Poinar JGO (2004). Mites, nematodes and the multimillion dollar weevil. Journal of Natural History.

[ref-22] Labandeira C (1998). How old is the flower and the fly?. Science.

[ref-23] Lajis NH, Hussein MY, Toia RF (1985). Extraction and identification of the main compound present in *Elaeis guineensis* flower volatiles. Pertanika.

[ref-24] Legendre P, Legendre L (1998). Numerical ecology 2nd English Edition.

[ref-25] Li K, Tscharntke T, Saintes B, Buchori D, Grass I (2019). Critical factors limiting pollination success in oil palm: a systematic review. Agriculture, Ecosystems and Environment.

[ref-26] Lubis FI, Sudarjat, Dono D (2017). Populasi serangga penyerbuk kelapa sawit *Elaeidobius kamerunicus* Faust. dan pengaruhnya terhadap nilai fruit set pada tanah berliat, berpasir dan gambut di Kalimantan Tengah, Indonesia. Jurnal Agrikultura.

[ref-27] Markow TA, O’Grady P (2008). Reproductive ecology of Drosophila. Functional Ecology.

[ref-28] McAlpine JF (1987). Manual of Nearctic Diptera Volume 2.

[ref-29] Moore D, Howard FW, Moore D, Giblin-Davis RM, Abad RG (2001). Insects of palm flowers and fruits. Insects on palms.

[ref-30] Oksanen J, Blanchet FG, Kindt R, Legendre P, Minchin PR, O’Hara RB, Simpson GL, Solymos P, Stevens MHH, Wagner H (2015). http://CRAN.R-project.org/package=vegan.

[ref-31] Perovic DJ, Gurr GM, Raman A, Nicol HI (2010). Effect of landscape composition and arrangement on biological control agents in a simplified agricultural system: a cost-distance approach. Biological Control.

[ref-32] Poinar GO, Jackson TA, Bell NL, Wahid MB-A (2002). *Elaeolenchus parthenonema* n. g. n. sp. (Nematoda: Sphaerularioidea: Anandranematidae n. fam.) parasitic in the palm-pollinating weevil *Elaeidobius kamerunicus* Faust, with a phylogenetic synopsis of the Sphaerularioidea Lubbock, 1861. Systematic Parasitology.

[ref-33] Prasetyo AE, Purba WO, Susanto A (2014). *Elaeidobius kamerunicus*: application of hatch and carry technique for increasing oil palm fruit set. Journal of Oil Palm Research.

[ref-34] Prasetyo AE, Susanto A (2012). Serangga penyerbuk kelapa sawit *Elaeidobius kamerunicus* Faust.: agresivitas dan dinamika populasi di Kalimantan Tengah. Jurnal Penelitian Kelapa Sawit.

[ref-35] R Core Team (2018). https://www.r-project.org.

[ref-36] Rahardjo BT, Rizali A, Utami IP, Karindah S, Puspitarini RD, Sahari B (2018). Population site of *Elaeidobius kamerunicus* Faust (Coleoptera: Curculionidae) on different age of oil palm plantation. Indonesian Journal of Entomology.

[ref-37] Rizali A, Clough Y, Buchori D, Hosang MLA, Bos MM, Tscharntke T (2013). Long-term change of ant community structure in cacao agroforestry landscapes in Indonesia. Insect Conservation and Diversity.

[ref-38] Sahari B (2012). Community Structure of Hymenopteran Parasitoid in Oil Palm Plantations, Pandu Senjaya Village, Pangkalan Lada District, Central Kalimantan (in Bahasa). Dissertation.

[ref-39] Strauss SY, Whittall JB, Harder LD, Barrett SCH (2006). Non-pollinator agents of selection on floral traits. Ecology and evolution of flowers.

[ref-40] Susanto A, Purba RY, Prasetyo AE (2007). Elaeidobius kamerunicus: Serangga Penyerbuk Kelapa Sawit.

[ref-41] Syed RA (1979). Studies on oil palm pollination by insects. Bulletin of Entomological Research.

[ref-42] Syed RA, Push-Parajah E, Chew PS (1982). Insect Pollination of Oil Palm: feasibility of Introducing *Elaeidobius* spp. [Species] into Malaysia [From Africa].

[ref-43] Teo TM (2015). Effectiveness of the oil palm pollinating weevil, *Elaeidobius kamerunicus*, in Malaysia. UTAR Agriculture Science Journal.

[ref-44] Tuo Y, Koua HK, Hala N (2011). Biology of *Elaeidobius kamerunicus* and *Elaeidobius plagiatus* (Coleoptera: Curculionidae) main pollinators of Oil Palm in West Africa. European Journal of Scientific Research.

[ref-45] Xu Z, Zhou G (2017). Identification and control of common weeds: volume 1–3.

[ref-46] Zuur AF, Ieno EN, Walker NJ, Saveliev AA, Smith GM (2009). Mixed effects models and extensions in ecology with R.

